# Distributed Coordination of Heterogeneous Agents Using a Semantic Overlay Network and a Goal-Directed Graphplan Planner

**DOI:** 10.1371/journal.pone.0062931

**Published:** 2013-05-21

**Authors:** António Luís Lopes, Luís Miguel Botelho

**Affiliations:** Instituto de Telecomunicações, Instituto Universitário de Lisboa (ISCTE-IUL), Lisboa, Portugal; Indiana University, United States of America

## Abstract

In this paper, we describe a distributed coordination system that allows agents to seamlessly cooperate in problem solving by partially contributing to a problem solution and delegating the subproblems for which they do not have the required skills or knowledge to appropriate agents. The coordination mechanism relies on a dynamically built *semantic overlay network* that allows the agents to efficiently locate, even in very large unstructured networks, the necessary skills for a specific problem. Each agent performs partial contributions to the problem solution using a new distributed goal-directed version of the Graphplan algorithm. This new goal-directed version of the original Graphplan algorithm provides an efficient solution to the problem of "distraction", which most forward-chaining algorithms suffer from. We also discuss a set of heuristics to be used in the backward-search process of the planning algorithm in order to distribute this process amongst idle agents in an attempt to find a solution in less time. The evaluation results show that our approach is effective in building a scalable and efficient agent society capable of solving complex distributable problems.

## Introduction

One of the major challenges of creating real-world agent societies is to develop a coordination infrastructure that is scalable and robust enough to support cooperation in increasingly complex problems. Multi-agent coordination has been the focus of much research in the area of distributed problem solving and multi agent systems. Although extensive research has been done in this area, most of that work relies on centralized or hierarchical structures. The work presented in this paper combines multi-agent coordination and peer-to-peer (P2P) computing as an efficient way of deploying scalable and efficient distributed problem solving. Our main goal is to build a coordination framework that allows heterogeneous agents to freely participate in totally decentralized large-scale collaborative environments. In the presented proposal, agents are able to use their own skills to partially contribute to complex problems and delegate the remaining unsolved subproblems to other agents that are better equipped to further contribute to the problem's solution.

The remainder of the paper is organized as follows. In the back section, we provide a background description of the problem we have addressed and the related work. This includes the description of our own work on the underlying discovery mechanism based on the dynamically built *semantic overlay network* (some of the concepts described here were presented in previous papers but we summarize them here, though in less detail, for the sake of making this paper self-contained) and the description of the planning graph paradigm and the *Graphplan* algorithm. In the dist section, we present our overall distributed approach to this coordination problem by first describing our distributed version of the *Graphplan* algorithm and then the goal-directed version of the previously described distributed algorithm. The evaluation section is dedicated to present and discuss the results of applying both versions of the algorithm to complex planning environments and, finally, in the conclusion section, we conclude the paper and discuss some limitations of our approach which will be considered in future research.

## Background

Even though classical planning approaches such as state-space planning [1], plan-space planning [2] and *graph planning* [3] have evolved to address fairly complex problems [4] [5] [6] [7], these are still inadequate to be used in large distributed environments, since they only consider the single agent view. In growing environments where multiple heterogeneous agents operate without a central or hierarchical structure, there is a need to efficiently coordinate the network of intelligent agents in order for their collective power to be used in providing solutions to complex problems.

Usually, current research addresses the coordination problem for distributed problem solving by making use of centralized components [8] [9] [10] or organizational structures [11] [12] [13] [14] [15] [16] [17] [18] that are potential central points of failure, may introduce inefficiencies, and may not scale to larger environments, which can compromise the entire system. Moreover, major comparisons of multi agent coordination strategies [19] [20] show that centralization is only suitable when the environment is composed of a few hundred agents and that distributed approaches are clearly more effective for larger networks.

Adopting a *divide and conquer* strategy to solve the multi agent coordination problem has proven to be an effective alternative [21]. The use of goal transformations [22] is an example of such a coordination mechanism. Basically, this strategy allows an agent, who has to solve goal G, to solve a goal G' instead that generates a sub-solution and then pass the remainder of the goal (i.e. G minus G') to another agent. In more detail, an agent looking to solve goal G must solve the set of open conditions of this goal, *i.e.*, it must have the necessary skills to solve all the desired conditions that are not true in the current state. If the agent does not have the necessary skills to do so, it divides the open conditions into two sets: one with conditions that the agent is capable of achieving; and another with the remaining conditions. It is this set of open conditions, for which the agent cannot contribute, that is sent to another agent, hoping it will contribute to satisfy them.

For the agent to know who to delegate this set of conditions it cannot cope with, the whole set of agents is organized in subdomains [22]. Each agent represents the sub-domain to which it belongs, which contains not only its own actions, but also a set of phantom actions it does not possess. A domain can be split into any number of sub-domains bounded by the total number of actions in the domain. Phantom actions point to the agents that possess them. Hence, if an agent does not own an action, it knows which agent to enlist for that action.

However, from a network topology point of view, this means that each agent must have a phantom connection to all other agents in the network (referred to as a *fully-connected* network), which is prohibitive for very large and dynamic networks with high churn rates (the rate at which agents enter or leave a network) making the approach non-scalable. In the experiments presented in [22], the authors have only used a maximum of 3 agents with a maximum of 3 actions each. The described mechanism concatenates the resultant sub-plans from agents into a final solution plan, without a central coordination process. However, it does not take into account possible conflicts that may arise from the fact that agents only contribute to parts of the problem without considering the effect that their decisions may have on other agents' contributions.

Although the approach described above does not rely on centralized components, it makes use of a distributed setting that is not scalable for large networks or for networks in which there is a high churn rate. We address the efficient management of large networks with high churn rates without using centralized elements, superimposed organizational structures or fully-connected networks. Using the semantic link paradigm to create meaningful connections amongst the agents in the environment (also referred to as *semantic overlay networks* [23]) is an effective way to optimize the discovery process. If, as shown in [24], an agent has a resource or a skill that is somehow related to a resource or skill that belongs to another agent, then it is important that a semantic based connection exists between these two agents stating the meaning of their relationship. This semantic-link can then be used to improve future searches or collaboration initiatives. This avoids having too many connections between the agents (as in fully-connected networks) since only semantically-related agents are connected.

### Semantic-based Distributed Discovery Mechanism

For an agent to contribute to solve a specific problem that can only be solved by the collective effort of different agents in a network, it needs an efficient discovery algorithm to find the agents that provide the other necessary contributions. Peer-to-peer (P2P) computing research has provided interesting solutions, namely informed searches [25] [26] [27] [28] [29] and distributed hash tables (DHT) [30] [31] [32]. However, these semantic-free approaches, although having good performance for point queries (like DHT), they are not as effective for approximate, range, or text queries [23] and they do not, on their own, capture the relationships between an action or agent's name and its content or metadata [33].

A semantic approach aims at bringing a more powerful and meaningful description of agents and their actions so as to optimize the discovery process in collaborative environments. In previous work [34] we have shown that it is possible to improve the resource coordination process in multi agent peer-to-peer networks by building, maintaining and using a *semantic overlay network* [23] that is dynamically learnt and updated by the discovery mechanism. The discovery and self-organization process, in which agents establish semantic connections amongst them, thus fueling the *semantic overlay network*, is first carried out by using efficient and robust search mechanisms and network evolution techniques (see [34] and [35] for details). The process in which the semantic overlay is built starts as soon as agents enter the network and broadcast their skills. This is an on-going process that is never actually complete since new agents can enter the network at any time. Agents can use the *semantic overlay network* to easily locate resources, for example, while performing other tasks such as planning a solution for a specific problem. When doing so, if an agent detects that a certain skill cannot yet be found in the semantic overlay network, it can trigger the discovery mechanism in order to update the semantic overlay network.

During this process, each agent uses a simple inference rule to determine whether or not other agents should be semantically linked to it: agent 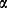
's action 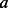 should be semantically linked to agent 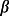
's action 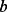 if 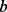
's effects (denoted as 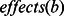) contribute to achieve 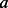
's preconditions (denoted as 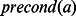). The following expression illustrates the inference rule (we consider that preconditions and effects are sets of propositions which represent their conjunction):
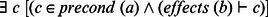
(1)


The main purpose of this process is to allow a network of otherwise unrelated agents to self-organize, such that each agent knows exactly where the actions on which its own actions depend (or contribute to) are. This information will be useful for coordinating agents within the distributed problem solving process.

Nevertheless, this process has to be efficiently carried out and should be flexible and scalable enough to support increasingly larger networks. We have developed two algorithms, Priority-based Flooding (PbF) and Iterative Branching Depth-First Search (IBDFS) and compared them with existing ones. Both these algorithms were presented and evaluated in [34] and [35]. In this paper, we briefly describe the best of the two algorithms, IBDFS, which is used in our coordination system.

Our proposed search technique, Iterative Branching Depth-First Search (IBDFS), is based on the depth-first search mechanism and it can be used as an alternative to other algorithms (even in high load networks). We introduce the use of an iterative branching process in the depth-first search to increase the coverage of the network. When initiating a search process, an agent will randomly contact one of its neighbors. If the neighbor possesses the required skill, then the agent informs the requesting agent and the process ends. If the neighbor replies stating that it does not have the required skill and that it will apply the same iterative branching depth first search process with its neighbors, then the agent will contact a second neighbor and so forth. Algorithm 1 depicts the recursive discovery algorithm.

**Algorithm 1** IBDFS(q, N): let *q* be the query to be processed, *N* the list of neighbors and *r* the result of a query processing event.



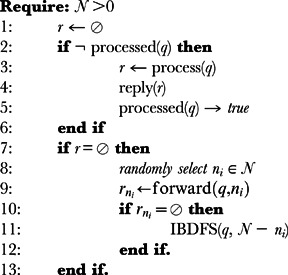



This approach increases the branching level iteratively on each hop count, thus increasing the chances of finding the answer faster, comparatively to the depth first search approach.

### Planning-graph and the Graphplan Algorithm

Given an initial state of the world and a goal or set of goals (we use the STRIPS [1] representation under the assumption of a deterministic and fully observable domain) and a set of potential actions, a *planning graph* [3] consists of a directed, leveled graph where levels alternate between proposition levels containing proposition nodes and action levels containing action nodes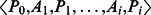. The first level (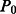) is a proposition level composed of proposition nodes corresponding to the initial state. The second level (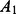) is an action level composed of action nodes, one for each action whose preconditions are satisfied by the propositions in the first level (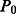). The third level (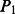) is a proposition level composed of proposition nodes, which represent the propositions created by the effects of the actions in the second level. Also, the propositions created by previous proposition levels are added to this level. That is, at each level 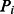, each proposition 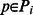 is propagated to the next level 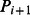 by a dummy action *no-op* that has a single precondition and a single positive effect 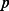.

The *planning graph* is built this way until a proposition level is reached that includes all propositions of the goal. A *planning graph* does not represent a valid plan for a planning problem. Instead, it uses the principles of *independence* and *mutual exclusion* - or *mutex* - to drastically reduce the search space and help finding a valid plan faster.

Two actions 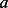 and 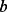 are independent if and only if the following two conditions hold (we denote 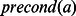 as the preconditions of an action 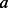, and respectively 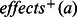 as the positive effects of 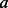 and 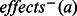 as the negative effects of 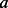):
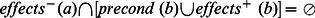
(2)

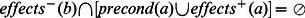
(3)


Two actions 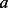 and 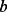 in level 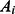 are mutex if either 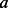 and 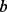 are dependent or if a precondition of 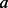 is mutex with a precondition of 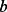. Two propositions 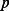 and 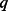 in 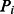 are mutex if every action in 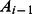 that has 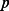 as a positive effect (including *no-op* actions) is mutex with every action that produces 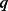. The set of 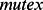 relations at proposition level 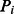 and action level 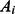 are denoted respectively 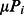 and 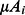.

*Graphplan* [3] is an example of how a *planning graph* can be used for solving a planning problem. The *Graphplan* algorithm iteratively expands the *planning graph* by one level (with the exception of the first expansion, which is done until a proposition level is reached where all goal propositions are included and no pairs of them are mutex since it does not make sense to start searching for a plan in a graph that has not yet reached a goal state) and then searches backward from the last level of this graph for a solution. The search procedure looks for a set of non-*mutex* actions that achieve the goal propositions. Preconditions of the chosen actions become the new goal propositions and the process continues. A failure to meet the goal at some level 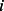 leads to backtrack over all other subsets of actions in level 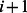. If the first level is successfully reached, then the corresponding action sequence is a solution plan.

This iterative graph expansion and search processes are pursued until either a plan is found or the search reveals that no solution can be found in the *planning graph*. A way to determine that a problem has no solution and to avoid an infinite expansion process is by using the *level-off* property, which dictates that every *planning graph* has a fixed-point level 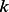 that is the smallest 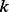 such that 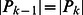 and 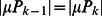.

*Graphplan* has revolutionized automated planning research mainly because of its simple, elegant algorithm and its representation of planning problems that created the basis for an extremely fast planner [36]. Nevertheless, the algorithm applies to the one-agent planning paradigm and does not explore the potential of using *Graphplan* in a distributed setting.

## Methods

If we interpret the problem to be solved as a goal (or a set of goals) to be achieved, then its decomposition into sub-problems requires the decomposition of the main goal into smaller sub-goals. Hence, agents need a planning algorithm that allows them to decompose the initial goal (or goals) into smaller goals, to contribute to achieve some of them and to delegate the remaining to other, more suitable, agents. We have built a distributed version of the *Graphplan* planner [3] that allows agents to do just that: perform local contributions to the current *planning graph* and delegate the partially filled graph to more suitable agents (which are discovered using a *semantic overlay network*).

However, as with similar *Graphplan*-based approaches [37] [38], its performance tends to degrade at an undesirable rate as problems grow in size or complexity. This is due to the problem of "*distraction*", in which planners using forward chaining algorithms waste a lot of time considering propositions in the initial state that are irrelevant to the final solution plan. In this paper we describe and evaluate the goal-directed version of our distributed *Graphplan* planner, which performs a preliminary graph expansion process that starts from the goal state and produces a special-purpose graph (called *operators-graph*) with only the planning operators (or actions) that are relevant to the problem at hand. Once this *operators-graph* is distributedly built, the normal expansion of the planning graph can then proceed using only the relevant operators found in the *operators-graph*. The final backward graph search, in which agents try to find a valid solution to the planning problem, cannot be distributed, but it can be tackled with different strategies. In this paper, we also discuss and evaluate a set of heuristics that allow a group of agents to perform this search using different techniques as a way of finding the solution as quickly as possible.

### Distributed Graphplan

In the centralized version of the Graphplan algorithm, the planning agent has full knowledge of the available actions. However, in a distributed environment, each agent only has knowledge of its own actions. We modified the algorithm to take into account partial contributions to the development of the *planning graph*. The process is carried out as follows (Algorithm 2 details the expansion process engaged by each agent):

**Algorithm 2** Expand(i, PG): Let i be the current level of expansion in the planning graph, I the set of propositions in the initial state, G the set of goal propositions, PG a *planning graph* with the structure 〈*P*_0_, *A*_1_, *µA*_1_, *P*_1_, *µP*_1_ …, *A_n_*, *µA_n_*, *P_n_*, *µP_n_*〉 and A the set of actions the agent knows (**Note :** the sets in the algorithm with a superscripted 2 in the name, represent all possible pairs of the elements of those sets):



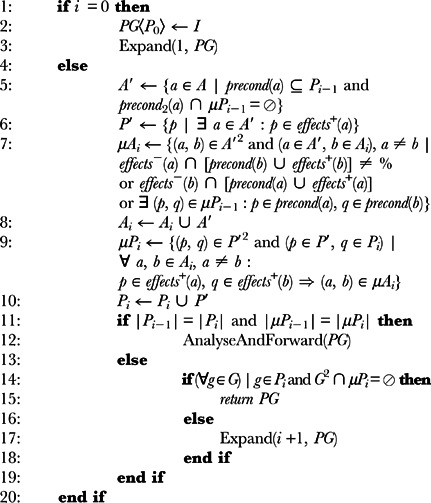

- An agent receiving a planning problem solving request, which includes a description of the initial state and a set of goals, creates the first proposition level (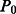 - line 2 of alg. 2) that is composed of all propositions of the initial state (this is only done by the first agent that receives this request);
- The agent then determines which of its own actions can be added to each action level 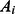 (line 5 of alg. 2) and corresponding propositions (actions' effects) to level 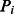 (line 6 of alg. 2) of the *planning graph*;
- *Mutexes* are calculated for all possible pairs of added actions and of those with the actions in level 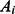. The *mutexes* between actions already present in level 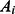 do not have to be recalculated. An identical process is carried out for propositions (see lines 7 and 9 of Algorithm 2 for details).
- When the agent is unable to make further contributions to the *planning graph* (i.e., when the *planning graph levels-off* - line 11 of the alg.), it analyses the open propositions (to which it was unable to contribute) and forwards the partial *planning graph* to an agent chosen from a set of *appropriate* agents (obtained using the agent discovery mechanism supported by the *semantic overlay network* - line 12 of alg. 2);
- The new agent receiving the *planning graph* will execute these same steps up to a point where a level 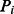 in the graph is reached where all goal propositions exist and none of which is mutex with any other (line 14 of alg 2), or until a certain terminating condition holds.

The AnalyseAndForward procedure in the algorithm (line 12) encapsulates the choice of the agent to which the partially-filled planning graph should be sent. There are several different ways as to how this process can be carried out and we provide a detailed analysis on this in the Results and Discussion section, in particular, the Open Conditions and Resolvers sub-section.

The termination of this overall expanding process in a distributed environment is not trivial. In the centralized version, an agent can declare that a problem is impossible, if the graph *levels-off*. For an agent with only partial knowledge of the world, it is impossible to know if a *leveled-off* graph means that the problem is impossible or if it simply means that the agent does not have the necessary skills to complete it and should, therefore, request the contribution of another agent.

This could lead to an indefinite process of forwarding partially solved problems between agents. To prevent this situation, we use a similar mechanism as the one used in P2P search algorithms, where a *time-to-live* (TTL) parameter is used to specify the allowed number of times the request may be forwarded without it being updated with new contributions. Once that TTL parameter expires, the problem is considered impossible and the requester agent is duly informed. Although this may seem to cause the algorithm to be incomplete, the Graphplan algorithm is in fact complete. It is only the inability of the discovery process to find the necessary skills (because these may not exist in the network or they would require several iterations through the network to be found) that causes the algorithm to not reach a solution plan.

Once a *planning graph* reaches a point where all goal propositions exist and none of which are *mutex*, it is up to the agent holding the *planning graph* at that time to execute the backward search (starting from the goal propositions) that will find a valid solution plan. The agent can also request the assistance of other agents in the backward search. In such cases, each agent will use a different heuristic in the process (an analysis of the heuristics used in the backward search is presented in the evaluation section, in particular, the eval:heuristics sub-section). Algorithms 3 and 4 carry out this whole process.

**Algorithm 3** Extract(PG, G, i): Let PG be a *planning graph* with the structure 〈*P*_0_, *A*_1_, *µA*_1_, *P*_1_, *µP*_1_ …, *A_n_*, *µA_n_*, *P_n_*, *µP_n_*〉, G the current set of goal propositions, i the current level being analyzed and *π_i_* a set of actions that achieve propositions of G:



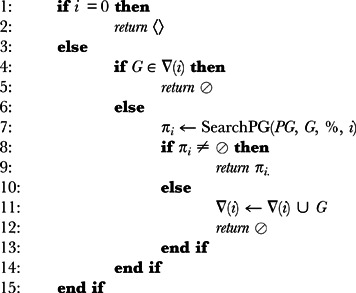



**Algorithm 4** SearchPG(PG, g, *π_i_*, i): Let PG be a *planning graph* with the structure 〈*P*_0_, *A*_1_, *µA*_1_, *P*_1_, *µP*_1_ …, *A_n_*, *µA_n_*, *P_n_*, *µP_n_*〉, G the current set of goal propositions, *π_i_* a set of actions that achieve propositions of G, i the current level being analyzed, *A_i_* the action level i, *µA_i_* the *mutexes* between actions in *A_i_* and Π the current solution plan:



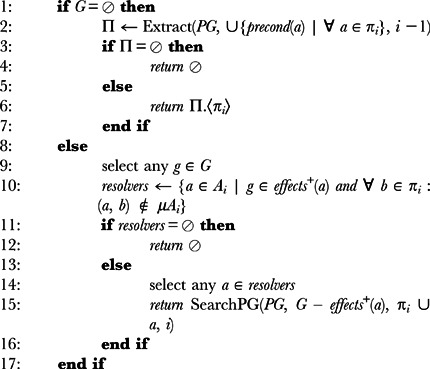



Algorithm 3 takes as input a planning graph, a current set of goal propositions and a current level index. It extracts a set of actions that achieves the goal propositions by recursively calling Algorithm 4 (line 7). If it succeeds in reaching level 0, then it returns an empty sequence (lines 1 and 2), from which pending recursions successfully return a solution plan.

The *mutex* relation between propositions provides only forbidden pairs, not tuples. But it might be the case that the search process shows that a tuple of more than two propositions corresponding to an intermediate sub-goal fails. To avoid analyzing the same (invalid) tuple more than once, which might occur due to the backtracking and the iterative deepening of the backward search process, algorithm 3 records any information regarding failed tuples (in the hash table denoted by 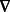 - in line 11) and checks each current goal with respect to these recorded tuples (in line 4) to save time in future searches.

Algorithm 4 selects each goal proposition p at a time (line 9) and from the resolvers of p, that is, actions that achieve p and that are not *mutex* with actions already selected for that level, it chooses one action a (line 14) that tentatively extends the current subset 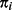 through a recursive call at the same level (line 15). This is performed on a subset of goals minus p and minus all positive effects of a in g. If a failure regarding this choice occurs, a backtrack over other alternatives for achieving p (if any) or a backtrack further up (if all resolvers of p have been tried) is performed. When g is empty (line 1), then 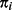 is complete. At this point, the search recursively tries to extract a solution for the following level i-1 (line 2). This process carries on until the first proposition level is reached successfully and a final solution plan is extracted from the planning graph.

### Goal-directed Distributed Graphplan

In most domains, some of the propositions contained in the initial state are completely irrelevant to reach the goal state of a specific problem. As most forward-chaining planners, *Graphplan* suffers from the problem of *distraction*, where the planner considers all propositions in the initial state even if they will not help reach a solution plan. In fact, these unnecessary propositions have an undesirable effect because they can be very time-consuming, thus degrading the performance of the planner. Therefore, they should be avoided. The problem lies in the fact that forward-chaining planners do not know which propositions are relevant.

To cope with this problem, we have used a similar approach to the one presented in [5]. We use *means-ends* analysis in the *Graphplan* algorithm, by first producing an *operators-graph* [39] using a backward-chaining process starting from the goal state. Since it only considers the propositions in the goal state, the *operators-graph* will produce a graph with only relevant actions.

This planner uses a similar process to the one used in the generation of the *planning graph* but in the opposite direction, as shown in Algorithm 5. It finds actions (including *no-ops*) that can contribute to goal propositions (line 5 of alg. 5) and the preconditions of those actions become the new goal propositions (line 6 of alg. 5).

**Algorithm 5** BuildOG(i, OG): Let *i* be the current level of expansion in the *operators-graph*, *G* the set of propositions in the goal state, *OG* an *operators-graph* with the structure 〈*P_n_*, *A_n_*, …, *P*_1_, *A*_1_, *P*_0_〉 and *A* the set of actions the agent knows:



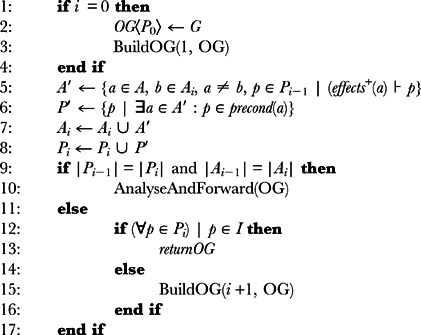



The process continues until it reaches a level in which all propositions are contained in the initial state (line 12 of alg. 5) or the graph *levels*-*off* (line 9 of alg. 5). Since there is no *mutexes* calculation in this process, the *level-off* property in this case is slightly different: the *operators-graph* has a fixed-point level 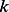 that is the smallest 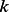 such that 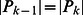 and 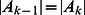.

Once the graph has been generated, the forward expansion of the *planning graph* (Algorithm 2) can take place, except this time it will only consider the operators that are contained in the *operators-graph*, thus significantly reducing the size of the graph and the number of *mutexes* calculations.

The generation of this graph is a lot faster because it is not as complex as the forward-based *planning graph* generation (which includes calculating *mutexes*). But since it does not analyze the relations between actions of the same level, it still generates actions that, even though relevant, may not occur in a solution plan. Nevertheless, this approach still presents advantages for domains in which the *distraction* problem has a strong impact, because it will consider a lot less actions than the original *Graphplan* algorithm. The drawback is, obviously, the overhead introduced by the generation of the *operators-graph*.

## Results and Discussion

As described in previous sections, we have developed two algorithms that allow agents to partially contribute to solve faced problems using only their limited knowledge of the world, enabling each agent to delegate the yet to be achieved goals to other agents, which may be discovered, relying on the *semantic overlay network* based P2P search algorithm. One of those algorithms is our distributed version of the *Graphplan* algorithm (see the Distributed Graphplan sub-section).

The other algorithm is a goal-directed version of the distributed *Graphplan* algorithm (see the Goal-directed Distributed Graphplan sub-section), which considers a lot less actions than the original approach but, in order to do so, it introduces a considerable overhead by having to generate the *operators-graph*.

This section presents the tests performed to evaluate these two algorithms. We begin by briefly presenting, in the eval:scenarios sub-section, the two scenarios in which the algorithms were extensively tested (a detailed description of these scenarios is available online at http://antoniolopes.info/files/appendices/scenarios). Then, in the Distribution of Skills sub-section, we evaluate the overall performance of the algorithms as the problems grow in size, in both previously described testing scenarios. These tests clearly show that the goal-directed version of the distributed *Graphplan* algorithm scales better than the other version of the algorithm, by simply employing a much more efficient planning graph generation process. In the Distribution of Skills sub-section we continue the analysis of the scalability and efficiency of the goal-directed version of the algorithm by evaluating and concluding that its performance is not affected by variations in the number of agents and skills in the environment. In the eval:ocr sub-section we explore a different strategy for choosing resolver agents and evaluate how this affects the performance of the system. Finally, in the eval:heuristics section we discuss and evaluate a set of heuristics for performing the backward search on the planning graph.

### Testing Scenarios

Our approach is intended to be used in environments where a problem, described as a set of goals to be achieved, must be solved through decomposition and delegation possibly to several agents. In such environments, agents have different capabilities, which may or not be complementary, and it is their collaborative work that ultimately produces a solution to the problem. In this sub-section we describe two such environments, the *Rescue Agents* and *Custom Balls Factory* scenarios, in which we have deployed and tested our approach:

- *Rescue Agents* - In this scenario, agents represent entities that participate in a rescue operation after the occurrence of a natural disaster, where they have to perform operations such as clearing roads, putting out fires and providing assistance to injured people.
- *Custom Balls Factory* - In this scenario, agents represent machines that can apply different types of customization in the production of sports balls, such as color, size, shape, fabric type, filing, manufacturing process and other properties.

The scenarios, which were chosen because they represent diverse large classes of coordination problems, are deliberately different to allow analyzing and testing different aspects of the coordination approach. On one hand, we have the *Rescue Agents* scenario, which in spite of the low number of different types of entities (paramedics, ambulances, firemen and policemen), is a very complex planning scenario due to the high level of interaction/cooperation that is needed between the agents. In almost any situation, all entities of the environment are required to intervene to provide the best assistance possible to the injured people, thus making conflicts management the top most priority of the planning activity. Basically, this scenario is intended to represent those coordination problems in which small teams of individuals have to intensely collaborate (to avoid conflicts) to solve very complex or large problems (which usually lead to very large solution plans), such as rescue operations, project planning or soccer-playing robots.

On the other hand, we have the *Custom Balls Factory* scenario, which in spite of involving many different capabilities, is a fairly simple planning scenario. For each manufactured ball, only a very small set of skills is needed from the vast selection of existing capabilities, thus characterizing this scenario as a discovery challenge. The planning process on this scenario only becomes relevant when the requested customization of the ball uses a set of interdependent features requiring a specific execution sequence (for example, a ball must first be fully painted with one color and only then can stripes be painted with another color - executing these actions in reverse order would result in the effects of the paint action canceling the effects of the stripes action). Basically, this scenario represents those coordination environments in which the problems to be solved are usually simple and small but for which the number of possible candidates to participate in the creation of the solution plan is huge, such as service coordination, travel planning or event planning.

### Overall Performance

First of all, we wanted to test and analyze the overall performance of the planning algorithms in both scenarios, as the problems became larger. We have performed a set of tests using increasingly complex variants of these scenarios on both algorithms. In the *Rescue Agents* scenario we used 3 different types of entities (paramedic, ambulance driver and fireman) and 10 agents for each of those entities. We then increased the number of injured people and the number of fires (there was one fire for the tests with 1–4 injured people and a new fire was introduced on the variants with 5 or more injured people) in the environment to test the performance evolution of the algorithms.

In the *Custom Balls Factory* scenario we used 20 combinations of different types of features of the balls manufacturing process (color, size and other distinct marks combined with painting, assembling and inflating) and 2 agents for each of those combinations. We then increased the complexity of the manufactured ball by changing the number of features of the ball and the dependencies between them.

**Figure 1:**
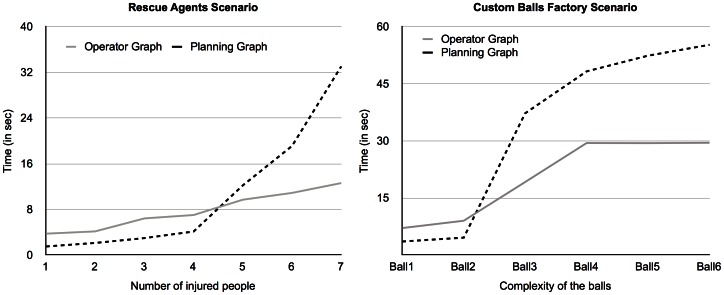
Comparison between the two algorithms in both testing scenarios. Comparison between Distributed Graphplan and Operators-graph-based Distributed Graphplan in both testing scenarios, with the *Rescue Agents* scenario on the left panel and the *Custom Balls Factory* scenario on the right panel.

Figure 1 presents the test results for both scenarios (left diagram for the *Rescue Agents* scenario and right diagram for the *Custom Balls Factory* scenario). The measured time represents the overall planning time, including the distributed graph generation (*operators-graph* - where applicable - and planning-graph) and backward search. The *semantic overlay network*'s generation time is not included since it is not of significance in the overall planning time (100–200 ms) and because it is an activity that agents perform as they connect to the network, which means the overlay network is already built when they receive the problem solving request.

Both scenarios' test results show a similar behavior: although the *operators-graph* based algorithm has poorer performance in smaller problems (when there are less injured people or the balls are less complex), it is clear that it scales far better than the distributed Graphplan algorithm. This is strongly linked to the fact that, for more complex or large problems, *means-ends* analysis is effective in reducing the planner search space, in spite of the introduced overhead. This is particularly evident in the *Rescue Agents* scenario.

The irregular behavior of the *operators-graph* based algorithm in the *Custom Balls Factory* scenario, apparent in the right diagram of fig. 1, is due to the fact that this scenario is more sensible to changes in planning complexity. As explained above, this scenario is more of a discovery challenge and, since the *semantic overlay network* is such an efficient agent discovery mechanism, as long as the number of conflicts between capabilities does not increase (e.g. due to ordering or dependency constraints), the performance remains the same. This is clear in the figure for balls 4, 5 and 6, which in spite of having a different number of features, the constraints between them are the same and thus, do not affect the performance of the system.

To fully understand what is really causing those behaviors in the algorithms, let us analyze a breakdown of the activities of each algorithm in the *Rescue Agents* scenario.

**Figure 2:**
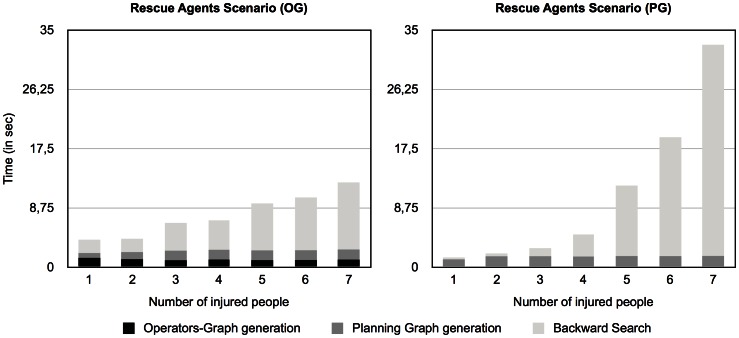
Comparison between algorithms in the ***Rescue Agents*** **scenario.** Breakdown and comparison of activities between Operators-graph-based Distributed Graphplan (left panel) and Distributed Graphplan (right panel) in the *Rescue Agents* scenario.

Figure 2 presents the breakdown of activity data for the same test as shown in the left diagram of fig. 1 but divided into two diagrams (the one on the left presents the *operators-graph* based algorithm and the one on the right presents the planning graph version that does not build the *operators-graph*).

As we can see, the most time-consuming activity is the Backward Search process. This is the task that involves searching the planning graph backwards in order to find a valid solution plan. The generation of the *operators-graph*, although causing poorer performance in simpler problems, is very efficient in improving the Backward Search phase in larger and more complex problems by significantly reducing the number of actions that are considered in the planning graph generation process (although not shown here, the same conclusions apply to the *Custom Balls Factory* scenario).

### Distribution of Skills

In the previous sub-section, we tested the behavior of the planning algorithms as the problems became larger in order to assess their scalability and efficiency. This showed that the *operators-graph* based version of the distributed *Graphplan* algorithm scales far better. However, the scalability and efficiency analysis must also assess planner behavior as the number of available agents (and corresponding skills) increases.

**Figure 3:**
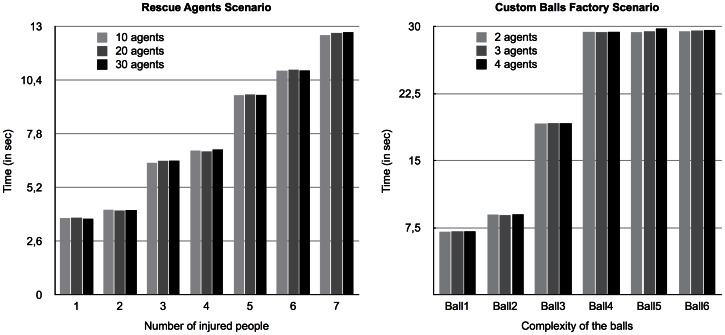
Evolution of the performance of the algorithm in both testing scenarios. Evolution of performance of the Operators-graph-based Distributed Graphplan in both testing scenarios (with the *Rescue Agents* scenario on the left panel and the *Custom Balls Factory* scenario on the right panel) as skills distribution increase. The given number of agents is per skill.

In the tests shown in the previous section, the number of agents per skill was 10 in the *Rescue Agents* scenario and 2 (per combination of skills) in the *Custom Balls Factory* scenario. The tests shown here, in Figure 3 (left diagram for *Rescue Agents* scenario and right diagram for *Custom Balls Factory* scenario), present the results for the same tests as in the previous section but with an increasing number of agents per skill (or combination of skills).

As we can see, in both scenarios, there seems to be almost no variation in the overall performance of the planner as the number of agents per skill increases. The lack of variation is caused by the fact that, when the time comes to choose an agent to which the partially-solved problem should be sent, even though the choosing agent now has a larger number of alternatives to consider, it chooses the appropriate agent randomly. Hence, the number of existing candidate agents is of no relevance to the performance of the planner.

**Figure 4:**
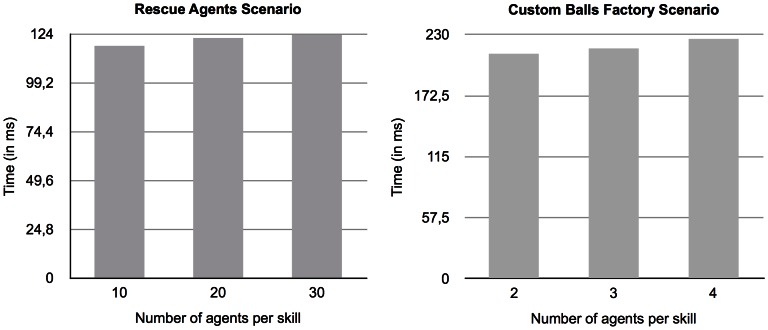
Comparison of time to generate the *semantic overlay network* as the number of agents per skill varies.

In the previous sub-section, we did not consider the time it took to generate the corresponding *semantic overlay network* for each scenario because it was equal for all the tests and it was too insignificant relatively to the overall planning time. However, now that the number of agents varies (and thus the *skill distribution factor*) the generation of the *semantic overlay network* is different for each test. We use the expression *skill distribution factor* as a measure of the amount of different skills existing in the network (relative to the total number of agents) and consequently their availability. Figure 4 depicts the time taken to generate the overlay network for each variation of the number of agents per skill, for both scenarios.

The figure depicts a very slight variation of the time to generate the *semantic overlay network* as the number of agents per skill varies. Although the number of agents has significantly increased (twice or three times more), the time it takes to generate the semantic network is almost unchanged because, as the number of agents increases, the *skill distribution factor* decreases and so, the more likely it is to find each different skill in the network. Hence, the variation of the time that it takes to complete the generation of the *semantic overlay network* in networks with more agents per skill is considerably smaller.

### Open Conditions and Resolvers

Each agent only plays a small part in the overall problem solving process. When the agent realizes that it can no longer contribute to the problem at hand, it must find a suitable agent that can potentially contribute to the unsolved sub-problems. As previously explained, the agent chooses one of the open conditions in the planning problem (propositions that remain unsatisfied in the current graph) and uses the semantic overlay network to determine which agents (and corresponding skills) can be used to further contribute to solve it. Once a list of candidate agents has been obtained, the agent must choose one to which the current problem will be forwarded.

**Figure 5:**
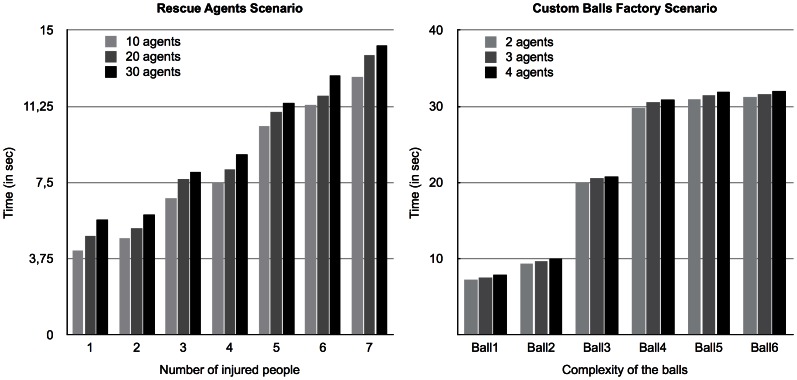
Evolution of performance of the algorithm in both testing scenarios (2). Evolution of performance of the Operators-graph-based Distributed Graphplan in both testing scenarios (with the *Rescue Agents* scenario on the left panel and the *Custom Balls Factory* scenario on the right panel) as skills distribution increase using a specific heuristic. The given number of agents is per skill.

Up to this point, in the tests performed to evaluate our approach, the agents made these decisions randomly. However, it is important to determine the influence a deeper or more sophisticated analysis of the open conditions and available resolver agents may have on the performance of the algorithm. One possible (and intuitive) approach is to quantify the contribution of each candidate agent by choosing the agent that can contribute to the largest number of open conditions in the current graph. We applied this heuristic to the planning algorithm and performed the same tests as in the previous sub-section, which results are shown in Figure 5.

We can see in the figure that the performance of the planner got worse as the number of agents per skill increased in both scenarios. However, the variation was smaller in the *Custom Balls Factory* scenario. This is related to the fact that each agent, when faced with the decision to choose the next agent to forward the planning graph, has to perform the same open conditions/skills analysis to a larger number of candidate agents. In the case of the *Custom Balls Factory* scenario, the number of candidate agents per combination of skill is much smaller than in the *Rescue Agents* scenario, therefore the performance of the algorithm is less affected in the *Custom Balls Factory* scenario.

This leads us to conclude that a random approach is, in general, more suitable for choosing the next open condition/agent to proceed in the planning process. Nevertheless, one cannot disregard the advantages of a more careful analysis such as the one depicted in this heuristic, just because, for these particular scenarios, the introduced overhead was too much to compensate for the gain in the performance of the planner. This is further discussed in the next section, in particular, the Choosing Appropriate Resolvers Based on Context sub-section.

### Heuristics in Planning Graph Backward Search

In the Distributed Graphplan section, we described the backward search process that is carried out to find a valid solution plan when the generation of the planning graph achieves a state in which all goal propositions are satisfied. This search process starts from the goal propositions and finds sets of non-mutex actions that contribute to those goals and then the preconditions of those actions become new goals (in the previous level). This process continues until the first level is reached successfully, in which case, pending recursions successfully return a solution plan.

Although the planning graph generation process is distributed, allowing different agents to contribute to the planning problem, this backward search process cannot be distributed. Moreover, sending the planning graph to other agents, so that these could also perform a backward search, would be pointless because they would simply be duplicating efforts.

However, as shown in Algorithm 4, this search process has two important choice points that may affect the performance of the search process: choosing a goal proposition (line 9 of alg. 4) and choosing an action resolver (line 14 of alg. 4). This could be used as a way to distribute the backward search through different agents as well.

Still, this would not be a "*divide and conquer*" approach. Instead, all agents would be working on the same planning graph but each one would be using a different heuristic. This can be thought of as a maze with multiple entrances. The path to the other side of the maze constitutes the solution plan. The goal is for at least one agent to find the solution, which it can then share with the others. If each agent starts at a different entrance, chances are they will arrive at the other side at different times because some paths take less time to travel than others.

The difference is that traveling through a maze is a totally uninformed task, whereas the algorithm for the backward search can be focused with heuristics for selecting the next proposition g in the current set G and for choosing the action a in resolvers. A general heuristic consists of selecting first a proposition g that leads to the smallest set of resolvers, that is, the proposition g achieved by the smallest number of actions. For example, if g is achieved by just one action, then g does not involve a backtrack point and it is better if it is processed as early as possible in the search tree. A symmetrical heuristic for the choice of an action supporting g is to prefer no-op actions first because they have fewer preconditions.

Other heuristics that are more specific to the planning-graph structure and more informed take into account the level at which actions and propositions appear for the first time in the graph. The later a proposition appears in the planning graph the most constrained it is. Hence, one would select the latest propositions first.

Considering all these possibilities, we decided to analyze the effect that different heuristics have on the performance of the planner, in particular, on the backward search process. We tested all possible combinations of heuristics for choosing a goal proposition and heuristics for choosing resolver actions. The following is a list of the heuristics for choosing a goal proposition:

- **FIFO** - priority to propositions that appear earlier in the graph;
- **LIFO** - priority to propositions that appear later in the graph;
- **Res
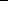
** - priority to propositions that have fewer action resolvers;
- **Res
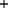
** - priority to propositions that have more action resolvers;
- **Random** - propositions are randomly chosen;

The following is a list of the heuristics for choosing a resolver action:

- **Precond
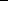
** - priority to resolvers that have fewer preconditions;
- **Precond
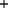
** - priority to resolvers that have more preconditions;
- **Random** - resolvers are randomly chosen;

The tests consisted on running the planner 100 times for each possible pair of heuristics on a simplified planning problem for the described scenarios. Table 1 presents the average results for all possible pairs of heuristics. Rows represent heuristics for choosing a resolver action and columns represent heuristics for choosing a goal proposition.

**Table 1:** Comparison of heuristics in *Graphplan* backward search process.

	FIFO	LIFO	Res^−^	Res^+^	Random
**Precond^−^**	45	**44**	240	5645	69
**Precond^+^**	90	49	230	8092	315
**Random**	67	52	242	6790	298

Comparison of heuristics in *Graphplan* backward search process. Values are in milliseconds and represent *only* the time spent in the backward search process.

The results seem to support the hypothesis presented above, that is, choosing resolver actions that have less preconditions (which has a lower impact if backtracking occurs) and choosing propositions that appear later in the graph (which are more constrained) has a very positive effect. That combination (Precond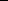 with LIFO) had the best time performance of all possible combinations (44 ms) and, in general, these individual heuristics combined with other heuristics (see row Precond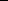 and column LIFO) have also presented satisfying results compared to other combinations. Processing the number of resolvers that a goal proposition has in order to choose the proposition with fewer resolvers (column Res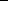), while it could apparently have a positive effect, the time spent determining the proposition with fewer resolvers is too much to actually bring any gain compared to the LIFO approach. Also, it is quite clear that using the opposite approach (column Res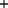) severely affects the performance of the search process, due to the "heavy" backtracking that is required to deal with giving priority to goal propositions that have more resolvers.

Random heuristics do not present any generic pattern, in some cases presenting good results and in others the worst results, which is consistent with the random choice of goal propositions and resolver actions and further proves the results are sound.

Considering these results, we have decided to use the Precond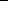 and Precond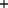 heuristics for choosing resolver actions and the FIFO and LIFO heuristics for choosing goal propositions. This way, each agent participating in a backward search process can use a different combination of heuristics. We also have to consider that these results may be different for more complex problems, which further motivates the use of different heuristics and the participation of different agents in the backward search for a solution plan.

## Conclusions

In this paper we have described the approach taken in a cooperative environment, where we have deployed a distributed network of problem solving agents by using a *semantic overlay network* and a distributed *Graphplan*-based algorithm. The evaluation results show that a goal-directed approach can be considered scalable and efficient. However, as most research, this is a continuous work and we aim to improve some of the aspects of the coordination system. In this section, we outline some of those aspects that need improvement, which will be the guidelines for future work.

### Exploring Alternative Solutions

Our approach focuses on finding the agents with the necessary capabilities in the network, as efficiently as possible, and performing the necessary planning to find a solution to each problem. However, finding just the necessary capabilities to solve a problem may end up producing inefficient solution plans. This is particularly important in time-sensitive scenarios such as *Rescue Agents* where it is essential that the entities in the environment act quickly to save the lives of the injured people.

Consider the following example: to rescue an injured person that is trapped inside some wreckage, our system would try to find a doctor and a fireman, which possess the necessary skills for the problem at hand. For this particular problem, this solution is, in fact, the optimal solution. However, imagine that there are, instead, several injured people and several doctors and firemen available. The system would still try to find only one doctor and one fireman (because that is enough to solve the problem) instead of creating the optimal solution plan that would explore the possibility of using several doctors and firemen in parallel.

However, improving our system to address this limitation is not an easy task. For example, imagine that an agent has already produced a solution plan for a specific problem but that the plan could be improved by adding other entities that could work in parallel to reach a potentially faster execution. This situation raises several questions. How can an agent know that the plan that it currently holds, although enough to solve the problem, can be improved by adding new participants? The only way the agent has to know for sure is to continue the collaboration process and continuously request the participation of new entities. But, if each agent is constantly assuming the solution plan can be improved by adding new participants, when does this process end? Maybe each agent can compare the resulting plans to determine if any improvements were actually made. If none were made, then the agent can assume the plan has reached an optimal state. Alternatively, each agent could perform the planning process with the goal of maximizing a domain-dependent evaluation function that would, for example, value plans with more parallelism.

However, without those domain-dependent functions, these agents can only operate with the partial knowledge of the skills available in the network. It might be the case that an agent is not able to further improve a solution plan, thus considering it to be optimal, but the plan could, in fact, be improved, if different capabilities were available to the agent. We are not aware of any way of ensuring that the best solution has been achieved in such distributed and decentralized environments. Moreover, in order to come up with potentially better solution plans, the distributed problem solving process must continue to explore new possibilities, which may result in much longer planning processes. In fact, we have performed a few preliminary tests in which the agents were forced to search for a better solution plan (until no further improvements could be made) and, while the solution plans were in fact better (less steps or more parallelism in the execution phase), the planning phase took a lot more time than our original approach. So, although it can potentially lead to a longer solution plan, our approach has the advantage of providing a much faster planning process.

### Acting on Behalf of Other Agents

Each agent in our system has only knowledge of its own skills. It is only after taking part in the self-organization process of building the *semantic overlay network* that an agent becomes aware of the skills of other agents, especially, of those semantically related to it. In our approach, this information is only used to locate agents that have the necessary skills to complete the solution to a particular problem. Once the skill is located, the agent currently holding the partially-solved problem sends it to the agent holding the required skill so that it can contribute to the plan.

What if, instead, the first agent used that information directly in its planning process thus saving the time it takes to communicate with the other agent? This has the potential of speeding up the planning process, but in doing so, the first agent is acting on behalf of the other agent in terms of commitment to participate in the solution plan. In other words, the first agent is assuming that, just because it has the necessary skills, the other agent will contribute to solve the given problem. This kind of assumption cannot be made because the first agent has no way of knowing if the second agent can commit to play the required role in the solution plan or if there are any constraints preventing it from doing so, for example, having previously committed to participate in another plan that would clash with this one.

Planning with such strategy would only lead to solution plans that most likely would not be executed due to the fact that the participating agents cannot perform the required actions because local constraints, which were not considered at planning time, prevent them from committing to the actions on the solution plan. A possible alternative is for agents to engage in a negotiation process in which they exchange constraints. For example, the first agent, before adding the action to the plan, would ask permission to the second agent, to which it could reply, after checking current local constraints, whether it accepts it or not. These messages are potentially less "expensive" from the communication's point-of-view because they are simple queries, compared to the size of the messages that are sent with partially-filled planning graphs. However, there may be more of them in quantity, which reduces the potential of this approach.

Agents cannot act on behalf of other agents unless they have their permission or they are aware of their constraints. In both cases, heavy communication may be required. However, once an agent is aware of other agents's constraints, it would no longer have to ask for them again (assuming these do not change over time and that the agent is in possession of all the constraints and not just a subset). This is not a safe assumption to make, especially in highly dynamic environments, but it may be of relevance for problems in which a continuous collaboration between two or more agents is required. For some agents, contributing to a solution plan only requires a small participation, that is, the number of times its actions appear in the final solution plan is quite small. However, in scenarios as the *Rescue Agents*, most participating agents have a more determining role in the solution plan, as a doctor having to provide assistance to 6 injured people located in different areas of a city. This problem, which requires the participation of a doctor and an ambulance driver, will continuously be sent back and forth between the two agents representing these two entities so that each can add its actions to the solution plan. A lot of communication can be saved if one of the agents simply performs the planning once all local constraints and necessary actions are known.

For example, consider that the first agent receiving the problem is the medic agent, which after processing it, determines that it can provide assistance to one of the injured people, but for that it requires the participation of an ambulance that can take him there. So, it contacts the ambulance agent by sending it the partially-solved problem. At this point, the ambulance agent already has the necessary knowledge to perform the entire planning process that would involve providing assistance to the remaining injured people. However, as explained before, it cannot commit to the plan on behalf of the medic agent unless it has its permission or it is aware of its constraints. But, if the medic agent, when sending the partially-solved problem to the ambulance agent, would also include its local constraints (as an implicit authorization to act on its behalf), then the ambulance agent could build the entire solution plan, thus saving a lot of messages in the process. We performed some preliminary tests and, in fact, the problems of the *Rescue Agents* scenario were solved in less time than in the original approach, whereas the problems in the *Custom Balls Factory* scenario had little or no improvement at all. Nevertheless, this approach, which is based on a potentially unsafe assumption that agents can commit to the plans on behalf of other agents (as long as they know their constraints), needs to be further analyzed.

An alternative approach could be based on abstract commitments at the planning stage that would only be realized at the execution stage. That is, an agent building the solution plan could include abstract commitments with the skills that it found on the network. These commitments are abstract in the sense that no actual agent has committed to them. They are only associated to a skill found in the network. Then, at the execution stage, agents with the necessary skills and without local constraints that would clash with the plan requirements perform those parts of the plan. We have worked on similar approaches before [40] [41] but further research is necessary to consider dynamic unstructured environments. This is something that we plan to do in the future.

### Choosing Appropriate Resolvers Based on Context

Each agent in this distributed problem solving process, after determining how its own skills can be used to partially contribute to the solution, must find a suitable agent that can potentially contribute to the unsolved parts of the problem. In most situations, this includes having to choose a particular agent from a long list of candidates, which may influence the performance of the system. We have used an approach in which the agent was chosen randomly from the candidates list, and later on discussed an alternative approach relying on an heuristic that would quantify the potential contribution of each candidate agent (see the Open Conditions and Resolvers sub-section).

The use of a random approach in choosing the adequate agent to contribute to the solution was justified simply by the fact that it was faster than choosing the agent that can solve more open conditions, in all performed tests. The random approach is faster because the overhead introduced by the heuristic approach was too much to compensate the improvement brought by its use. However, in more complex environments, such as the ones in which agents commit and act based on costs and rewards, a random approach can be very inefficient, leading to very costly solution plans. In such cases, the challenge revolves around identifying the information that should be used to select the appropriate agent.

The quantifiable contributions and the costs and rewards associated with the commitment of chosen agents are important to evaluate potential candidates, but other different sources of information can also be useful. Information such as the agent's general availability, workload, location and past average performance are just a few examples of contextual data that, in combination with other relevant data, can be used to narrow down the list of potential candidates.

Combining all of these considerations into a unified context-aware system is quite a challenge, but it is one in which we have already presented some promising research work [40] [41] [42]. We intend to evaluate how a context-aware based process can be used in such distributed unstructured environments to improve the process of choosing the appropriate resolvers for partial contributions in distributed problem solving.
